# High Serum IgA Identifies Poor Responders to Immune Checkpoint Inhibitors in Patients with Unresectable Esophageal Squamous Cell Carcinoma

**DOI:** 10.1245/s10434-026-19879-5

**Published:** 2026-05-30

**Authors:** Kosaku Torii, Akimitsu Iizuka, Dai Shimizu, Koki Nakanishi, Chie Tanaka, Nobuhiko Nakagawa, Shinichi Umeda, Yusuke Sato, Haruyoshi Tanaka, Hideki Takami, Norifumi Hattori, Masamichi Hayashi, Mitsuro Kanda

**Affiliations:** Department of Gastroenterological Surgery, Nagoya University Graduate School of Medicine, Nagoya, Japan

**Keywords:** Immune checkpoint inhibitor, Chemotherapy, Esophageal squamous cell carcinoma, Immunoglobulin A, Predictive biomarker

## Abstract

**Background:**

Immune checkpoint inhibitors (ICIs) have become a standard first-line treatment for unresectable esophageal squamous cell carcinoma (ESCC). However, a substantial proportion of patients have early disease progression. Reliable pretreatment biomarkers capable of predicting ICI efficacy are urgently needed. Immunoglobulin A (IgA) has been implicated in shaping an immunosuppressive tumor microenvironment, yet its clinical relevance in ESCC remains unclear.

**Methods:**

Serum IgA levels were measured in patients who had unresectable ESCC treated with first-line ICI-based therapy (*n* = 20) and compared with those in disease control subjects (non-ICI [*n* = 16] vs resectable ESCC [*n* = 26]) and healthy control subjects (*n* = 58). Associations between pretreatment serum IgA levels, survival times, and clinicopathologic variables were analyzed. The patients in each cohort were stratified into high- and low-IgA groups using cohort-specific median values.

**Results:**

Serum IgA levels were significantly higher in all the ESCC patient groups than in the healthy control subjects. In the ICI-treated group, high pretreatment serum IgA was significantly associated with shorter progression-free survival (hazard ratio, 9.26; 95% confidence interval, 1.96–43.7; *P* = 0.005). No correlation between serum IgA and prognosis was observed in the non-ICI and resectable ESCC groups, indicating ICI-specific relevance. Pretreatment serum IgA did not correlate with clinicopathologic factors, including tumor markers and immune-related adverse events.

**Conclusions:**

High pretreatment serum IgA may serve as a novel, minimally invasive biomarker predicting poor response to ICI therapy in unresectable ESCC. Serum IgA assessment could support treatment decision-making and patient stratification in this era of expanding immunotherapy use.

**Supplementary Information:**

The online version contains supplementary material available at https://doi.org/10.1245/s10434-026-19879-5.

Esophageal cancer remains one of the gastrointestinal malignancies with a poor prognosis, accounting for approximately 600,000 new cases and 500,000 deaths annually worldwide.^1^ Esophageal squamous cell carcinoma (ESCC), the predominant histologic subtype in East Asia and parts of Southern and Eastern Africa, is frequently diagnosed at advanced stages and demonstrates a high rate of recurrence even after curative resection.^2,3^ In Japan, current clinical practice guidelines recommend the use of immune checkpoint inhibitors (ICIs) as first-line therapy for unresectable ESCC, in combination with chemotherapy or radiotherapy.^4^

Several ICIs have demonstrated efficacy in phase 3 clinical trials as first-line treatments for unresectable ESCC, including anti–PD-1 antibodies (nivolumab and pembrolizumab) administered with chemotherapy^5^ and the combination of nivolumab with the anti–CTLA-4 antibody ipilimumab.^6^ However, these agents do not provide universal benefit across all patients. Early disease progression after the initiation of ICI therapy has emerged as a major clinical concern,^7^ underscoring the urgent need for reliable biomarkers capable of predicting therapeutic response before treatment.

To date, several biomarkers have been proposed as predictors of ICI efficacy, including programmed death-ligand 1 (PD-L1) expression (evaluated using the combined positive score [CPS] or tumor proportion score [TPS]), microsatellite instability (MSI) and mismatch repair (MMR) status, tumor-infiltrating lymphocytes (TILs) (particularly CD8-positive T cells), and tumor mutational burden (TMB).^8,9^These assessments, however, require immunohistochemical analysis or genomic profiling using tumor tissue, which entails several limitations: the need for biopsy or surgical specimens, variability related to sample preservation, high cost, prolonged turnaround time, and potential sampling errors due to intratumoral heterogeneity.^10,11^ A minimally invasive, low-cost biomarker that can be measured easily and repeatedly using blood samples (a liquid biopsy) would therefore be ideal.^12^

Immunoglobulin A (IgA) is well recognized as the principal antibody mediating mucosal immunity in the gastrointestinal and respiratory tracts, although approximately 10% of IgA circulates in the bloodstream and contributes to systemic immune responses.^13^ Elevated serum IgA levels have been observed in infectious diseases, chronic inflammatory conditions,^14^ and multiple myeloma.^14,15^ However, few studies have examined the relationship between serum IgA and systemic therapy for solid tumors, and reports linking serum IgA to the efficacy of ICIs are particularly scarce. To date, no study has investigated the association between serum IgA levels and ICI treatment outcomes in ESCC.

This study evaluated the association between pretreatment serum IgA levels and survival outcomes for patients with unresectable ESCC receiving first-line therapy to determine whether serum IgA could serve as a novel biomarker predictive of response to ICI-based treatment.

## Materials and Methods

### Ethics

This study adhered to the ethical guidelines outlined in the World Medical Association Declaration of Helsinki (2013) Ethical Principles for Medical Research Involving Human Subjects and received approval (approval no. 2014-0044) from the Institutional Review Board of Nagoya University (Nagoya, Japan). Written informed consent for the use of clinical samples and data was obtained from all patients in accordance with institutional review board requirements.

### Serum Samples from Cancer Patients and Healthy Volunteers

Among patients who received first-line systemic therapy incorporating immune checkpoint inhibitors (ICIs) (ICI plus chemotherapy or an ICI doublet) for unresectable ESCC between January 2022 and March 2025, 20 patients with pretreatment blood samples adequately preserved for IgA measurement were included as the primary analysis cohort (ICI-treated group). Two additional groups were analyzed as disease control subjects: 16 patients who had unresectable ESCC treated with non-ICI regimens (chemotherapy alone or chemotherapy plus radiotherapy) were categorized as the non-ICI group, and 26 patients with resectable ESCC who underwent neoadjuvant chemotherapy with DCF (docetaxel, cisplatin, and fluorouracil) followed by curative esophagectomy were categorized as the resectable group. Patients with comorbid conditions that could potentially affect serum IgA levels, including IgA nephropathy, chronic liver disease, and active autoimmune diseases, were not present in this study cohort.

**Fig. 1:**
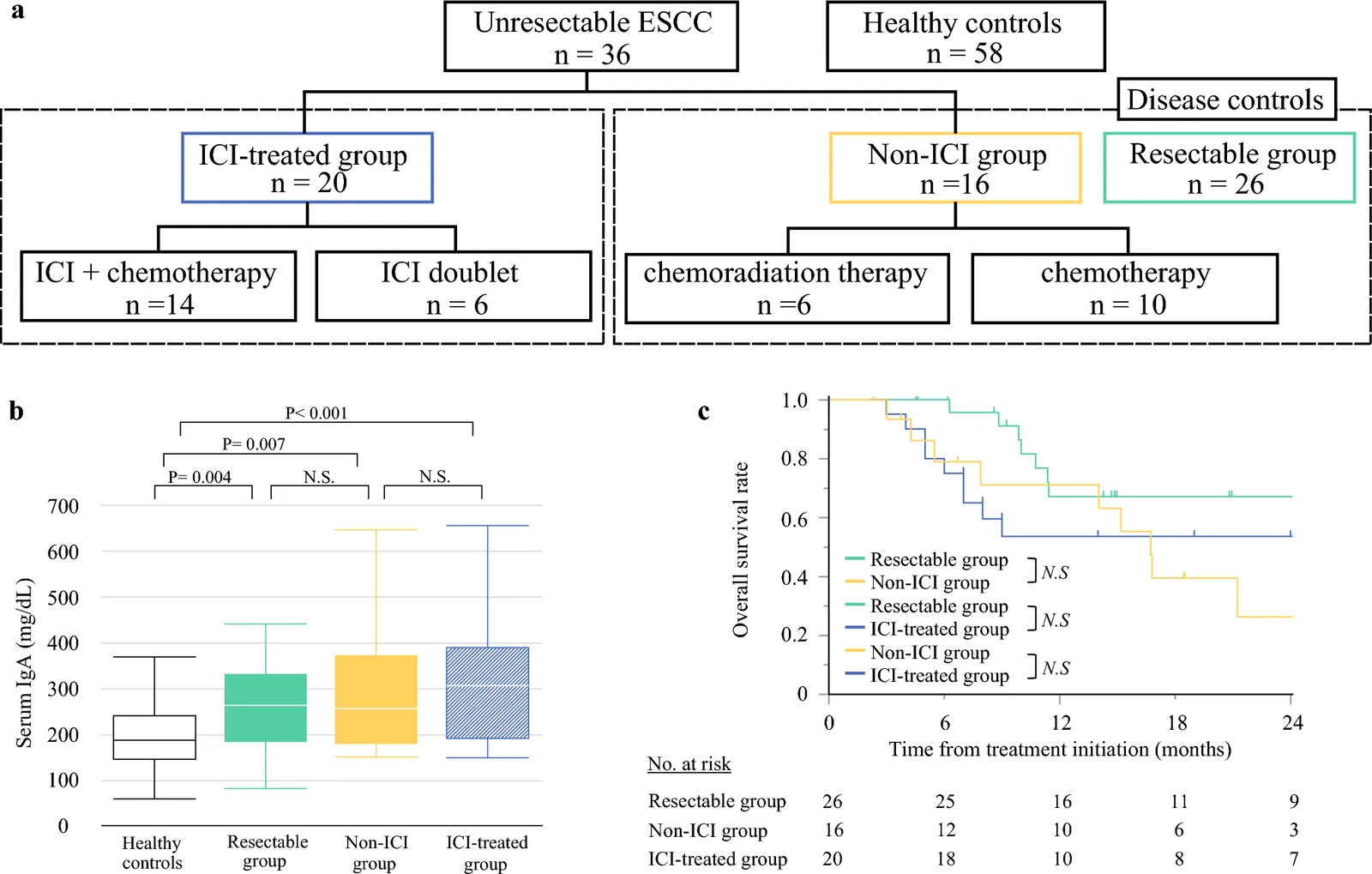
Patient grouping, serum immunoglobulin A (IgA) levels, and survival outcomes. **a** Flowchart illustrating the classification of subjects into the healthy control group, the immune checkpoint inhibitor (ICI)-treated group, the non-immune checkpoint inhibitor (ICI) group, and the resectable esophageal squamous cell carcinoma (ESCC) group. **b** Comparison of baseline serum IgA levels among the ESCC patient groups relative to healthy control subjects. **c** Kaplan–Meier overall survival curves for each group

Healthy adults (*n* = 58) who underwent annual medical examinations, including physical examination, blood testing, and chest radiography, at Nagoya University Hospital and who were not under active medical treatment at the time of enrollment served as the healthy control group. The analyzed cohorts are summarized in Fig. 1a.

Serum samples (1 mL) were collected from volunteers or cancer patients before initiation of treatment (within 7 days before the start of radiotherapy or systemic therapy). Blood was drawn into tubes containing serum-separating agents and maintained at 4 °C for at least 30 min. Samples were subsequently centrifuged at 3000 rpm for 5 min, and sera were transferred to fresh tubes and stored at −80 °C until analysis.

### Enzyme-Linked Immunosorbent Assay (ELISA)

Serum IgA concentrations were measured using the Human IgA ELISA Kit (ab196263, Abcam, Cambridge, UK) according to the manufacturer’s instructions. From each sample, 10 μL of serum was collected and diluted 2 × 10^5^-fold with the supplied dilution buffer. To each well, 50 μL of diluted sample and 50 μL of antibody cocktail (detection antibody, enzyme-conjugated antibody, and antibody diluent) were added and incubated at room temperature for 1 h. The wells were washed with 350 μL of wash buffer and allowed to stand for 10 s. Next, 100 μL of TMB development solution was added to each well and incubated in the dark for 10 min, after which 100 μL of stop solution was added and the plate was agitated on a shaker for 1 min to terminate the reaction. Absorbance was measured at 450 nm using a microplate reader (Multiskan GO; Thermo Scientific, Waltham, MA, USA). Standards were assayed in duplicate. A standard curve was generated over the range of 0.78–50 ng/mL, and the minimum detection sensitivity was 0.25 ng/mL.

### Immunohistochemical Analysis of Tumor Tissues

Immunohistochemical (IHC) analysis was performed using formalin-fixed paraffin-embedded (FFPE) tumor tissues obtained from 20 patients in the ICI-treated group according to the manufacturer’s instructions. Tumor specimens were collected via surgical resection or pretreatment biopsy (for non-surgical cases). Briefly, 4-μm-thick sections were deparaffinized in xylene and rehydrated through a graded series of ethanol. Antigen retrieval was performed by Tris-EDTA buffer (pH 9.0). Endogenous peroxidase activity was blocked with 3% hydrogen peroxide. The sections were then incubated with a primary antibody against IgA (clone 3A5A5, 60099-1-Ig; Proteintech Group, Rosemont, IL, USA) at a dilution of 1:10000 overnight at 4 °C. Subsequently, the sections were treated with peroxidase-labeled polymer secondary antibody and visualized using 3,3'-diaminobenzidine (DAB). Finally, the slides were counterstained with hematoxylin, dehydrated, and mounted. Human tonsil tissue sections were used as positive controls for IgA staining. The IgA expression was scored as follows; 2+ (strong staining in the ESCC component), 1+ (weak staining in the ESCC component), and 0 (no staining).

The IHC-stained slides were evaluated independently by two blinded observers to eliminate bias. To minimize intra-observer variation, each observer performed scoring at least twice. The IHC scores across both assessments were finalized. For slides with inconsistent scores, a third assessment was performed, and the score agreed upon by at least two of the three assessments was designated as the final score. In cases for which consensus was not reached, a third blinded observer independently scored the slides, and the final score was determined based on agreement by at least two of the three observers.

### Factors Analyzed

Serum IgA levels before initiation of treatment were compared between healthy control subjects and each ESCC treatment group. Tumor response and the best percentage change in target lesion size were evaluated according to Response Evaluation Criteria in Solid Tumors (RECIST), version 1.1, in the ICI-treated group. Associations between pretreatment serum IgA levels and progression-free survival (PFS), overall survival (OS), and various clinicopathologic factors also were assessed. We also investigated the relationship between pretreatment serum IgA levels and the intensity of IgA expression in tumor tissues.

### Statistical Analysis

Differences in serum IgA levels between healthy control and treatment groups were evaluated using the Mann–Whitney U test. Group means of categorical and continuous variables were compared using the chi-square test. Survival analyses were performed using the Kaplan–Meier method. Hazard ratios (HRs) were calculated using the Cox proportional hazards model, which also was used for univariate regression analysis. Progression-free survival was defined as the interval from treatment initiation to the date of progressive disease (PD) or death from any cause. Treatment response was evaluated by computed tomography (CT) every 2–3 months according to RECIST version 1.1. Overall survival included all causes of death, and subjects lost to follow-up evaluation were censored. Correlation between the IHC scores of IgA in tumor tissues and pretreatment serum IgA levels was evaluated using Spearman’s rank correlation coefficient. A two-sided *P* value lower than 0.05 was considered statistically significant. Statistical analyses were performed using JMP Student Edition 18 (SAS Institute, Cary, NC, USA).

## Results

### Serum IgA Levels in ESCC Patients and Healthy Volunteers

The median serum IgA level was 187 mg/dL (range, 60.0–369 mg/dL) in the healthy control group, 263 mg/dL (range, 82.4–671 mg/dL) in the resectable group, 256 mg/dL (range, 151–645 mg/dL) in the non-ICI group, and 308 mg/dL (range, 149–655 mg/dL) in the ICI-treated group. Pretreatment serum IgA levels in the resectable, non-ICI, and ICI-treated groups all were significantly higher than those in the healthy controls, but no significant differences were observed among the three ESCC patient groups (Fig. 1b).

### Characteristics of the ICI-Treated Group

The patient characteristics of the ICI-treated group are summarized in Table 1. An ICI plus chemotherapy regimen (pembrolizumab plus fluorouracil and cisplatin) was administered to 14 patients. Those who completed six cycles of cisplatin were subsequently transitioned to fluorouracil plus pembrolizumab or pembrolizumab monotherapy. Six patients received an ICI doublet regimen (nivolumab and ipilimumab). The primary reason for unresectability was distant metastasis in 13 patients. The median follow-up duration was 9 months (range, 2.3–40.3 months). Overall survival curves for the resectable, non-ICI, and ICI-treated groups are presented in Fig. 1c.

**Table 1:** Characteristics of the ICI-treated group (*n* = 20)

Variables	Values
Median age: years (range)	70 (42–77)
Sex	
Male	15
Female	5
Location	
Cervical, upper thoracic	3
Middle thoracic, lower thoracic	17
Differentiation	
Differentiated	13
Undifferentiated	7
Reason for unresectability	
Distant metastasis	13
T4	7
Disease status	
Postoperative recurrence	17
Initially unresectable	3
Treatment regimen	
ICI + chemotherapy^a^	14
ICI doublet^b^	6
Median serum IgA: mg/dL (range)	308 (152–655)

ICI, immune checkpoint inhibitor; IgA, immunoglobulin A

^a^Pembrolizumab + fluorouracil + cisplatin

^b^Nivolumab + ipilimumab

### Survival Analysis in the ICI-Treated Group

Analyses were performed for the ICI-treated group, which served as the primary focus of this study. The optimal cutoff value for serum IgA was determined by receiver operating characteristic (ROC) curve analysis using 6 month PFS as the endpoint. The optimal cutoff was 301 mg/dL (area under the curve [AUC], 0.79). Patients were subsequently classified into high-IgA (>301 mg/dL) and-low IgA (≤301 mg/dL) groups. No significant associations were observed between pretreatment serum IgA levels and clinicopathologic variables, including age, sex, Brinkman index, disease status, occurrence of immune-related adverse events (irAEs), serum carcinoembryonic antigen (CEA) levels, and serum squamous cell carcinoma-related antigen (SCC) levels (Table 2).

**Table 2:** Correlation of baseline serum IgA level with clinicopathologic factors in the ICI-treated group

Variables		Serum IgA level (*n* = 20)		*P* Value
		High (*n* = 10)	Low (*n* = 10)	
Age (years)	< 65 ≥ 65	4 6	3 7	0.639
Sex	Male Female	8 2	7 3	0.606
Brinkman index	≤ 500 > 500	4 6	4 6	1.000
Tumor location	Cervical, lower thoracic Middle thoracic, upper thoracic	2 8	1 9	0.531
Tumor differentiation	Differentiated Undifferentiated	6 4	7 3	0.639
Reason for unresectable	Distant metastasis T4	7 3	6 4	0.639
Disease status	Postoperative recurrence Initially unresectable	8 2	9 1	0.531
irAEs	Occurrence Non-occurrence	9 1	7 3	0.264
CEA (ng/ml)	≤ 5 > 5	7 3	7 3	1.000
SCC (ng/ml)	≤ 1.5 > 1.5	6 4	5 5	0.653

IgA, immunoglobulin A; ICI, immune checkpoint inhibitor; irAEs, immune-related adverse events; CEA, carcinoembryonic antigen; SCC, squamous cell carcinoma-related antigen

**Fig. 2:**
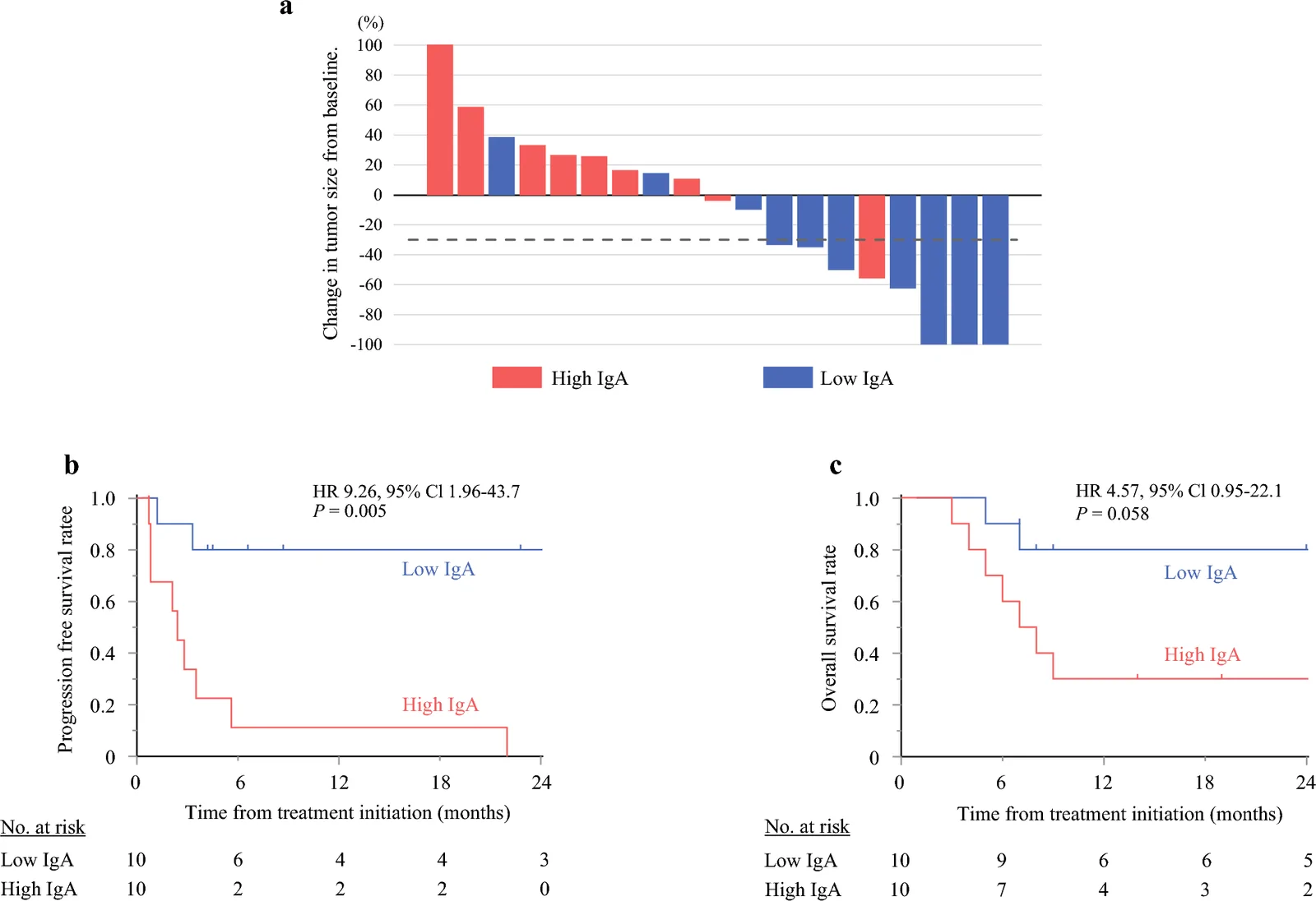
**a** Best percentage change in target lesion size from baseline according to RECIST version 1.1 in the immune checkpoint inhibitor (ICI)-treated group. Kaplan–Meier analysis of (**b**) progression-free survival and (**c**) overall survival in the ICI-treated group stratified by high or low serum immunoglobulin A (IgA) levels based on the median value. HR, hazard ratio; CI, confidence interval

The overall response rate was 10% in the high-IgA group and 70% in the low-IgA group (Table 3). Tumor responses to ICI therapy in the ICI-treated group are illustrated using a waterfall plot (Fig. 2a). One of the 20 patients was classified as having progressive disease due to the development of peritoneal dissemination and was therefore excluded from response evaluation. Tumor response was assessed in the remaining 19 patients with measurable lesions.

**Table 3:** Response rate for all patients and patients with high/low pretreatment serum IgA

	All patients (*n* = 20) *n* (%)	High IgA (*n* = 10) *n* (%)	Low IgA (*n* = 10) *n* (%)
Tumor response			
Overall response rate	8 (40)	1 (10)	7 (70)
Complete response	3 (15)	0 (0)	3 (30)
Partial response	5 (25)	1 (10)	4 (40)
Stable disease	5 (25)	2 (20)	3 (30)
Progressive disease	7 (35)	6 (60)	1 (10)

IgA, immunoglobulin A

Progression-free survival was significantly shorter in the high-IgA group than in the low-IgA group (HR, 9.26; 95% confidence interval [CI], 1.96–43.7; *P* = 0.005) (Fig. 2b). The 6 month PFS rate was 10% in the high-IgA group and 80% in the low-IgA group. In the univariate analysis for PFS in the ICI-treated cohort, high serum IgA was the only variable identified as a significant risk factor (Table S1). Regarding OS, the high-IgA group showed a trend toward shorter OS than the low-IgA group (HR, 4.57; 95% CI, 0.95–22.1; *P* = 0.058; Fig. 2c).

### Clinical Courses of Representative Cases in the Low- and High-IgA Groups

**Fig. 3:**
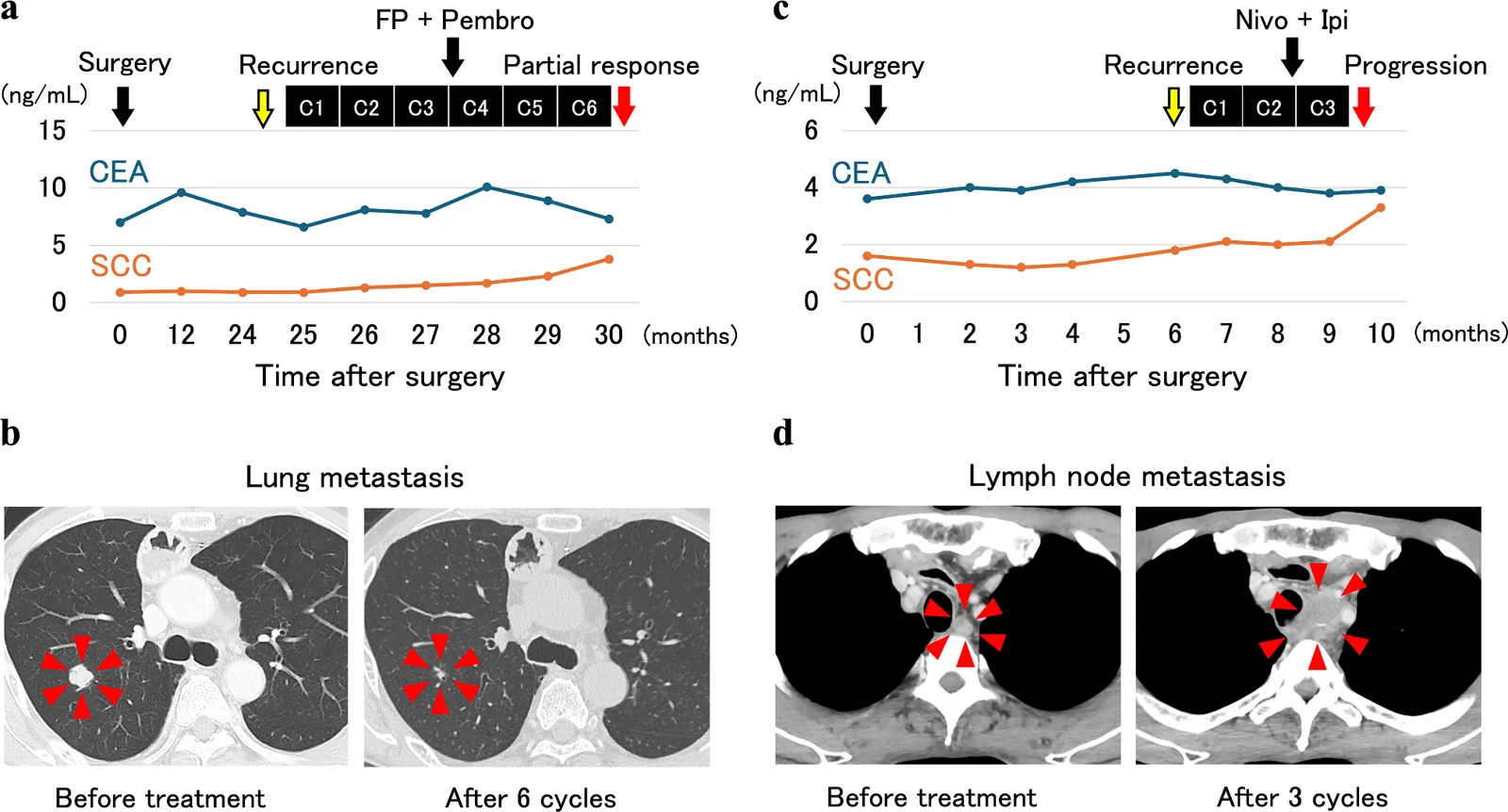
Longitudinal clinical courses and radiologic changes in two representative cases of the immune checkpoint inhibitor (ICI)-treated group. **a** Clinical timeline of case 1 showing treatment interventions and corresponding changes in tumor marker levels. **b** Contrast-enhanced computed tomography (CT) images of case 1 obtained at baseline and after two treatment cycles. The recurrent lesion (*arrows*) decreased markedly in size after treatment. **c** Clinical timeline of case 2 showing treatment interventions and corresponding changes in tumor marker levels. **d** Contrast-enhanced CT images of case 2 obtained at baseline and after three treatment cycles. The recurrent lesion (*arrows*) increased markedly in size after treatment. FP/Pem, fluorouracil plus cisplatin and pembrolizumab; Nivo/Ipi, nivolumab and ipilimumab

Among the cases analyzed in this study, two representative patients are presented. The first patient had low pretreatment serum IgA levels and showed a favorable response to ICI therapy. Conversely, the second patient had markedly elevated serum IgA levels (higher than the upper quartile) and experienced early disease progression. Figure 3 shows the CT findings before and after ICI initiation, together with longitudinal changes in tumor marker levels for both patients.

*Case 1:* A man in his 50s with a diagnosis of middle thoracic ESCC (cT1bN0M0, cStage I) underwent robot-assisted subtotal esophagectomy with two-field lymphadenectomy. The pathologic diagnosis was pT1bN1M0 (pStage IIA). He completed two cycles of adjuvant chemotherapy with fluorouracil and cisplatin and remained under postoperative surveillance. At 2 years after surgery, CT showed a left lung metastasis, for which treatment with fluorouracil, cisplatin, and pembrolizumab was initiated. The pretreatment serum IgA level was 152 mg/dL, categorizing the patient into the low-IgA group. After six cycles (5 months from treatment initiation), follow-up CT demonstrated a reduction in the size of the lung metastasis, which was evaluated as a partial response (PR) according to RECIST version 1.1. At this writing, the patient is continuing pembrolizumab monotherapy. No irAEs were observed during treatment.

*Case 2:* A man in his 50s with multifocal thoracic ESCC (cT1N0M0, cStage I) underwent robot-assisted subtotal esophagectomy with two-field lymphadenectomy. The pathologic diagnosis was pT1bN0M0, stage I. At 6 months postoperatively, CT showed recurrence in the lymph node at the upper mediastinal area, and ICI doublet therapy (nivolumab and ipilimumab) was initiated. The pretreatment serum IgA level was 407 mg/dL, corresponding to that of the high-IgA group. At 3 months after treatment initiation, CT examination showed enlargement of the metastatic lymph node and the appearance of new liver metastases. The patient exhibited disease progression and was switched to second-line therapy. No irAEs were observed during treatment.

### Analysis in Disease Control Groups

To determine whether the observed association between high pretreatment serum IgA and poor PFS was specific to ICI therapy or a general feature of systemic treatment, analyses were performed in the disease control cohorts (resectable group and non-ICI group). The patient characteristics for these groups are summarized in Table S2. For each disease control cohort, the patients were stratified into high- and low-IgA groups based on the median serum IgA value of the respective cohort. Unlike the ICI-treated group, pretreatment serum IgA levels were not associated with PFS in either the resectable or the non-ICI group (Fig. S1).

### Correlation Between IgA Expression in Tumor Tissues and Serum IgA Levels

**Fig. 4:**
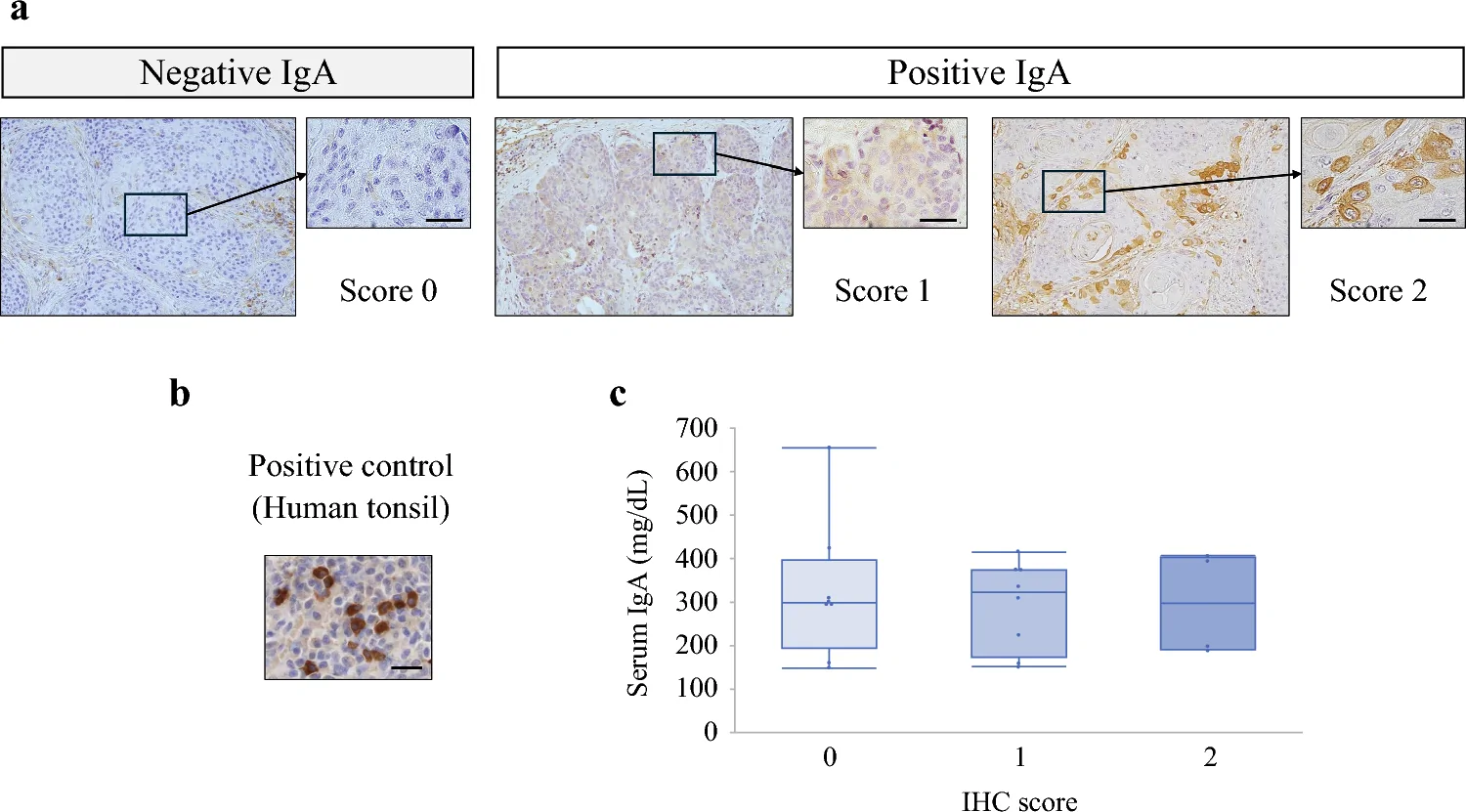
Intratumoral immunoglobulin A (IgA) expression and its correlation with pretreatment serum IgA levels. **a** Representative images of immunohistochemical (IHC) staining for IgA expression for each score (0, 1, and 2). Magnification: ×200 (*main images*) and ×1,000 (*insets*). Scale bars: 50 μm. **b** Human tonsil tissue used as a positive control. Scale bars: 50 μm. **c** Distribution of serum IgA levels according to intratumoral IgA expression scores (0, 1, and 2)

Figure 4 shows representative immunohistochemical staining patterns of IgA, displaying positive and negative expression in tumor tissues together with a positive control for IgA staining. The relationship between pretreatment serum IgA levels and IgA expression in tumor tissues was investigated. However, no significant correlation was observed between these two parameters (*ρ* = 0.05; *p* = 0.850). Consequently, our study did not demonstrate that systemic serum IgA levels directly reflect the local expression of IgA within the tumor tissues themselves.

## Discussion

This study investigated the association between pretreatment serum IgA levels and clinical outcomes, including prognosis and clinicopathologic variables, to evaluate their potential as predictive biomarkers for ICI efficacy in first-line treatment of unresectable ESCC. In the ICI-treated group, the overall response rate was 10% in the high-IgA group and 70% in the low-IgA group, and high pretreatment serum IgA levels were significantly associated with shorter PFS. In contrast, no association was observed between serum IgA levels and prognosis in either the resectable group or the non-ICI group, suggesting that this relationship is specific to ICI therapy.

Peppas et al.^16^ performed a meta-analysis of serum IgA levels in solid tumors and reported that IgA concentrations were significantly higher in head and neck cancer, breast cancer, and lung cancer than in healthy individuals. In esophageal cancer, a previous study by Saito et al.^17^ in 1992 demonstrated that serum IgA levels increased with advancing disease stage. Consistent with these findings, serum IgA levels in our ESCC cohorts were higher than in healthy controls. Increasing evidence also indicates that IgA-producing B cells and circulating IgA contribute to immune regulation within the tumor microenvironment (TME).^18^ In addition to serving as antibodies with direct cytotoxic activity, IgA-producing B cells express immunosuppressive molecules such as interleukin-10 (IL-10), transforming growth factor-beta (TGF-β), and programmed death-ligand 1 (PD-L1), which may suppress CD8⁺ T cell function and facilitate tumor immune evasion.^19^

Immune checkpoint inhibitors (ICIs) exert antitumor activity primarily by blocking the interaction between PD-1 on effector T cells and PD-L1 on tumor cells, thereby restoring cytotoxic T lymphocyte (CTL) activity.^20^ The ICI resistance observed in the high-IgA group suggests the presence of alternative suppressive pathways that interfere with CTL reactivation. Studies have shown that IgA-producing B cells secrete high levels of IL-10 and TGF-β, which are potent immunosuppressive cytokines that inhibit CTL proliferation and effector cytokine production, including interferon gamma (IFN-γ).^19,21^ Under sustained antigenic stimulation and suppressive cytokine exposure, T cells may enter a dysfunctional state known as exhaustion.^21^ Exhausted T cells exhibit persistent expression of inhibitory receptors, including PD-1, and demonstrate markedly reduced cytotoxic activity.^21^ Thus, a TME enriched with IgA-producing B cells may generate a cytokine milieu conducive to CTL exhaustion, thereby diminishing the therapeutic effects of PD-1/PD-L1 blockade. Supporting this concept, studies in hepatocellular carcinoma have shown that higher proliferative activity of peripheral CTLs correlates with improved response to pembrolizumab,^22^ underscoring the importance of CTL functional status in determining ICI efficacy. Collectively, elevated serum IgA levels may reflect an immunosuppressive environment that suppresses CTL activity, contributing to reduced responsiveness to ICI therapy.

In this study, we investigated the correlation between pretreatment serum IgA levels and intratumoral IgA expression to explore the potential link between systemic IgA and the TME. However, no significant association was observed. Several factors might explain this result. First, intratumoral heterogeneity may prevent IHC from fully capturing the overall IgA production within the TME. Second, there was a temporal gap between the collection of tumor tissues (at the time of surgery) and serum samples (at the time of recurrence or before ICI treatment). Consequently, although the theory that elevated serum IgA reflects an IgA-producing B cell-driven immunosuppressive TME is biologically plausible, it remains inferential and speculative based on our current data. Further longitudinal studies using matched tissue and serum samples are required to directly demonstrate this mechanism.

Reports on the association between serum IgA and ICI efficacy remain limited. In clear cell renal cell carcinoma, a recent study evaluating combination ICI plus tyrosine kinase inhibitor therapy demonstrated that responders (with partial or complete response) experienced a significant decline in serum IgA levels during treatment.^23^ However, that study did not show prognostic value for pretreatment IgA levels. In cervical cancer, Gong et al.^24^ reported that high pretreatment serum IgA levels were significantly associated with worse OS for patients receiving ICI therapy. They speculated that higher peripheral CD8⁺ T cell levels were associated with improved ICI responsiveness, suggesting that elevated IgA may indirectly reflect an immunosuppressive TME that impairs CTL function.

In our analysis, high pretreatment serum IgA levels were significantly associated with shorter PFS in the ICI-treated group. Notably, although the sample size was limited, the low-IgA group exhibited a favorable 2 year PFS exceeding 80%. Because serum IgA levels did not correlate with known prognostic factors, pretreatment serum IgA may represent an independent biomarker for predicting ICI efficacy. Serum IgA measurement offers several advantages over tissue-based biomarkers such as CPS or MSI, including its minimally invasive nature, low cost, accessibility using routine blood samples, and feasibility for repeated assessment. In this study, PD-L1 CPS was evaluable in only 7 (35%) of the 20 patients, which limited the power of our statistical analysis regarding the correlation between serum IgA levels and established tissue-based biomarkers. This limitation reflects the retrospective nature of the study and current clinical practice in Japan, where CPS testing is not routinely performed for all ESCC patients. Consequently, we could not determine whether serum IgA acts as an independent predictor or a complementary factor to PD-L1 expression. Future studies with larger, well-characterized cohorts are essential to clarify the relationship between serum IgA and existing biomarkers and to define its role in the treatment algorithm.

In Japan, ICI-based systemic therapy currently is recommended in clinical guidelines as a first-line treatment for unresectable ESCC.^4^ Because ICIs are increasingly incorporated into routine clinical practice, determining whether to continue or resume ICI therapy after irAEs is experienced remains a challenging clinical decision. In our study, no association was observed between pretreatment serum IgA levels and the development of irAEs. For cases in which irAEs occur, a low pretreatment serum IgA level, suggesting a higher likelihood of ICI benefit, may support the clinical decisions to continue or re-initiate ICI therapy.

This study had several limitations. First, because ICIs were only recently approved as a standard of care for ESCC in Japan (2022), the sample size was small and the follow-up period was relatively short. Although we confirmed that our cohort was representative of the general patient population by comparing OS outcomes (Fig. 1), the small sample size of 20 patients remained a significant limitation. This may have reduced statistical power, potentially leading to an overestimation of the observed effect sizes. Therefore, these results should be interpreted with caution. Furthermore, we assessed only pretreatment serum IgA levels. Investigating post-treatment changes in IgA concentrations and their correlation with clinical responses remains to be done in a future investigation. Only IgA was measured in this study, and IgG, IgM, complement components, and other immune-related factors were not evaluated. Future research incorporating a more comprehensive immunologic profile may further clarify the role of IgA in tumor immunity.

## Conclusion

High pretreatment serum IgA levels may serve as a novel biomarker associated with reduced efficacy of first-line ICI therapy for patients with unresectable ESCC.

## Supplementary Information

Below is the link to the electronic supplementary material.

Supplementary file1 (TIFF 36745 KB)

Supplementary file2 (DOCX 16 KB)

Supplementary file3 (DOCX 17 KB)
