# Supplementary material for: High Serum IgA Identifies Poor Responders to Immune Checkpoint Inhibitors in Patients with Unresectable Esophageal Squamous Cell Carcinoma
Source: Ann Surg Oncol. 2026 May 30;33(10):8900–9. doi: 10.1245/s10434-026-19879-5 (PMC13585957; doi:10.1245/s10434-026-19879-5)
Supplement: Supplementary file 1 [file 10434_2026_19879_MOESM2_ESM.docx]

**Supplementary TABLE 1**　Univariate analyses of progression free survival in the ICI-treated group

| Variables | n | Univariate analysis | | |
| --- | --- | --- | --- | --- |
|  |  | HR | 95% CI | *P* |
| Age (≥ 65) | 13 | 0.73 | 0.23-2.33 | 0.598 |
| Sex (Male) | 15 | 0.80 | 0.22-2.98 | 0.741 |
| Brinkman index (> 500) | 12 | 0.82 | 0.24-2.82 | 0.750 |
| Tumor location (Cervical; lower thoracic) | 3 | 0.51 | 0.07-4.02 | 0.525 |
| Tumor differentiation (Undifferentiated) | 7 | 0.73 | 0.22-2.45 | 0.508 |
| Reason for unresectable (Distant metastasis) | 13 | 0.99 | 0.30-3.33 | 0.992 |
| Disease status (Postoperative recurrence) | 17 | 0.58 | 0.12-2.74 | 0.495 |
| irAEs (Non-occurrence) | 16 | 3.32 | 0.42-26.0 | 0.254 |
| CEA (> 5 ng/ml) | 6 | 0.93 | 0.25-3.45 | 0.916 |
| SCC (> 1.5 ng/ml) | 9 | 1.16 | 0.37-3.61 | 0.798 |
| Serum IgA (> median) | 10 | 9.82 | 2.11-45.8 | 0.004 |

HR, hazard ratio; CI, confidence interval; irAEs, immune-related adverse events; CEA, carcinoembryonic antigen; SCC, squamous cell carcinoma-related antigen; IgA, immunoglobulin A
