# Supplementary material for: High Serum IgA Identifies Poor Responders to Immune Checkpoint Inhibitors in Patients with Unresectable Esophageal Squamous Cell Carcinoma
Source: Ann Surg Oncol. 2026 May 30;33(10):8900–9. doi: 10.1245/s10434-026-19879-5 (PMC13585957; doi:10.1245/s10434-026-19879-5)
Supplement: Supplementary file 2 [file 10434_2026_19879_MOESM3_ESM.docx]

| **Resectable group** |  |  | **Non-ICI group** |  |
| --- | --- | --- | --- | --- |
| Variables | Values |  | Variables | Values |
| Median age (range) -year | 65(53-77) |  | Median age (range) -year | 67(55-75) |
| Sex |  |  | Sex |  |
| Male | 21 |  | Male | 14 |
| Female | 5 |  | Female | 2 |
| Location |  |  | Location |  |
| Cervical, lower thoracic | 4 |  | Cervical, lower thoracic | 1 |
| Middle thoracic, upper thoracic | 22 |  | Middle thoracic, upper thoracic | 15 |
| Differentiation |  |  | Differentiation |  |
| Differentiated | 21 |  | Differentiated | 15 |
| Undifferentiated | 5 |  | Undifferentiated | 1 |
| Clinical T factor |  |  | Reason for unresectable |  |
| T1-2 | 5 |  | Distant metastasis | 11 |
| T3-4 | 21 |  | T4 | 5 |
| Clinical N factor |  |  | Disease status |  |
| N0 | 1 |  | Postoperative recurrence | 16 |
| N1-3 | 25 |  | Initially unresectable | 0 |
| Number of DCF cycles |  |  | Treatment regimen |  |
| 1-2 | 2 |  | Chemotherapy | 10 |
| 3 | 24 |  | Chemotherapy + radiotherapy | 6 |
| Median serum IgA (range) - mg/dL | 263(82.4-671) |  | Median serum IgA (range) - mg/dL | 256(151-645) |

**Supplementary TABLE 2**　Patient characteristics of disease control groups (non-ICI group; n = 16 and resectable group; n = 26)

DCF, docetaxel, cisplatin, and fluorouracil; IgA, immunoglobulin A
